# Two-Photon Excited Fluorescence of NADH-Alcohol Dehydrogenase Complex in a Mixture with Bacterial Enzymes

**DOI:** 10.3390/biom13020256

**Published:** 2023-01-30

**Authors:** Ioanna A. Gorbunova, Maxim E. Sasin, Dmitry V. Yachkov, Denis A. Volkov, Alexei D. Vedyaykin, Andrey A. Nikiforov, Oleg S. Vasyutinskii

**Affiliations:** 1 Ioffe Intstitute, St. Petersburg 194021, Russia; 2 Institute of Nanobiotechnologies, Peter the Great St. Petersburg Polytechnic University, St. Petersburg 195251, Russia; 3 Institute of Cytology, Russian Academy of Sciences, St. Petersburg 194064, Russia

**Keywords:** NADH, PLP, dehydrogenases, fluorescence, TCSPC technique, intracellular biological fluorophores

## Abstract

Thorough study of composition and fluorescence properties of a commercial reagent of active equine NAD-dependent alcohol dehydrogenase expressed and purified from *E. coli* has been carried out. Several experimental methods: spectral- and time-resolved two-photon excited fluorescence, sodium dodecyl sulfate–polyacrylamide gel electrophoresis, fast protein liquid chromatography, and mass spectrometry were used for analysis. The reagent under study was found to contain also a number of natural fluorophores: free NAD(P)H, NADH-alcohol dehydrogenase, NADPH-isocitrate dehydrogenase, and pyridoxal 5-phosphate—serine hydroxymethyltransferase complexes. The results obtained demonstrated the potential and limitations of popular optical methods as FLIM for separation of fluorescence signals from free and protein-bound forms of NADH, NADPH, and FAD that are essential coenzymes in redox reactions in all living cells. In particular, NADH-alcohol dehydrogenase and NADPH-isocitrate dehydrogenase complexes could not be optically separated in our experimental conditions although fast protein liquid chromatography and mass spectrometry analysis undoubtedly indicated the presence of both enzymes in the molecular sample used. Also, the results of fluorescence, fast protein liquid chromatography, and mass spectrometry analysis revealed a significant contribution of the enzyme-bound coenzyme pyridoxal 5-phosphate to the fluorescence signal that could be separated from enzyme-bound NADH by using bandpass filters, but could effectively mask contribution from enzyme-bound FAD because the fluorescence spectra of the species practically overlapped. It was shown that enzyme-bound pyridoxal 5-phosphate fluorescence can be separated from enzyme-bound NAD(P)H and FAD through analysis of short fluorescence decay times of about tens of picoseconds. However, this analysis was found to be effective only at relatively high number of peak photon counts in recorded fluorescence signals. The results obtained in this study can be used for interpretation of fluorescence signals from a mixture of enzyme-bound fluorophores and should be taken into consideration when determining the intracellular NADH/FAD ratio using FLIM.

## 1. Introduction

All prokaryotic and eukaryotic cells exhibit intrinsic fluorescence from natural chromophores: tryptophan and other aromatic amino acids, flavin mononucleotide (FMN), reduced nicotinamide-adenine dinucleotide (NADH), its phosphate NADPH, oxidized flavine-adenine dinucleotide (FAD), elastin, collagen, etc. [1,2]. Some of these chomophores: FAD, NADH, collagen, and elastin emit within overlapping fluorescent bands in green and blue spectral range [3,4,5] giving the main contribution to cellular and tissue autofluorescence.

The fluorescence properties of FAD and NAD(P)H in solutions and cells were extensively studied during the last decades because of their important role in cytoplasmic and mitochondrial redox reactions [6,7,8,9]. Nowadays label-free fluorescence lifetime imaging (FLIM) allows to monitor metabolism in living cells and tissues and has already been applied for studying of metabolic changes in pathological cells in vitro [10,11,12] and in vivo [13,14]. Additional benefits of increased spatial resolution, penetration depth, and the ability to measure multiple fluorophores simultaneously can be obtained by combining FLIM with 2-photon excitation [14,15,16,17]. Sharick et al. [18] used multiphoton FLIM for characterisation of relative concentrations of several metabolic NAD(P)H-dependent enzymes in solutions and cells. They demonstrated that measured fluorescence decay times can be used to distinguish NAD(P)H bound to various metabolic enzymes in solutions, and to quantify changes in enzymes activities. Very recently Gorbunova et al. [19] reported different dependence of NADH and FAD quantum yields on solution viscosity and polarity.

However, thorough interpretation of NAD(P)H-FLIM images recorded in cells and tissues still remains the main challenge. It is known that there are more than 300 various NAD(P)H-containing enzymes in cells that can contribute to FLIM signals. Yaseen et al. [14] revealed that it is almost impossible to establish direct relationships between specific intracellular enzymes and resolvable fluorescence lifetime components of NADH and NADPH. Nevertheless, it has been shown [14] that modulation of distinct pathways of glycolysis and oxidative metabolism alters uniquely the relative proportion and total amounts of four time-resolvable NADH fluorescence components when compared to baseline metabolic activity. Most of the NAD(P)H-FLIM studies dealt with a double-exponential fluorescence decay characterized by two decay times: one of them of about 0.4 ns was associated with free NADH and another of about 2 or more nanoseconds was associated with enzyme-bound NAD(P)H [11,20]. As known [21] the fluorescence spectrum and lifetime of NADPH are identical to those of NADH in solution and therefore fluorescence from free NADH and NADPH molecules cannot be discriminated on the basis of their fluorescence lifetimes. Using genetic and pharmacological approaches to perturb NADH and NADPH metabolism in cells, Bracker et al. [11] reported that FLIM differentiates quantitatively between these two coenzymes. They observed [11] that bound NADH and NADPH possessed the decay times of 2.7 ns and 3.6 ns, respectively and suggested an approach for determination of the NADPH/NADH ratio. NAD(P)H fluorescence decay times were shown to be highly sensitive to coenzyme surroundings and conformational changes in various cells [17]. Using the phasor plot analysis Leben et al. [20] created a systematic framework by measuring fluorescence decay times of NADH and NADPH bound to various enzymes in solutions. They concluded [20] that the fluorescence decay times of free and enzyme-bound NAD(P)H are also influenced by other factors such as refractive index, solvent polarity, pH, and ion concentration.

Moreover, the analysis of FLIM raw data remains a challenge because full autofluorescence signals from cells contain contributions from both NADH and NADPH as well as from other natural fluorophores with overlapping absorption and fluorescence spectra. The interpretation of these spectra is often a great problem. Direct attempts to resolve this problem by using multiexponential models for FLIM analysis [12,14] met the necessity to take into consideration the numerical instability of these models especially in complex microenvironments in living cells [20]. Cao et al. [15] presented a technique for acquiring of NAD(P)H and FAD signals by using one- and two-photon excitation schemes simultaneously and optimization of emission spectral filters. Chorvat and Chorvatova [22] developed a new setup for cell fluorescence microscopy based on spectrally resolved time-correlated single photon counting (TCSPC) technique.

In spite of many researches already carried out more detailed studies of reference systems of pure NAD-dependent enzymes are needed for correct interpretation of FLIM data.

In this paper we present a thorough study of the fluorescence properties of NADH bound to alcohol dehydrogenase (ADH) in solutions in a mixture with other enzymes. The studies were carried out using a wide range of experimental methods: spectral and time-resolved analysis of fluorescence spectra under two-photon excitation as a function of excitation wavelength, sodium dodecyl sulfate–polyacrylamide gel electrophoresis (SDS-PAGE), fast protein liquid chromatography (FPLC), and mass spectrometry (MS) analysis. The enzymatic mixture under study was a commercial reagent of active equine ADH expressed and purified from *E. coli* that contained also a number of unknown enzymes. Our analysis revealed that the fluorescence spectra contained major contributions from free NADH, NADH-ADH and NADPH-isocitrate dehydrogenase (ICDH) complexes as well as pyridoxal 5-phosphate (PLP)- serine hydroxymethyltransferase (SHMT) complex. As shown, the combination of spectral and time-resolved analysis of the fluorescence was mandatory for separation the contributions from different emitting compounds, however only some of them could be resolved by pure fluorescence methods. In particular, the NADH-ADH and NADPH-ICDH complexes could not be optically distinguished in our experimental conditions although the FPLC and MS analysis undoubtedly indicated the presence of both NADH- and NADPH-containing enzymes in the molecular sample. At the same time our results suggest that the decay times analysis of time-resolved fluorescence allows to separate PLP-SHMT from NADH-enzyme and FAD-enzyme complexes, although the fluorescence spectra of PLP-SHMT and FAD-enzyme complexes practically overlap each other. Therefore the contribution from bound PLP to fluorescence signal could mask contribution from FAD that should be taken into consideration in FLIM studies where the NADH/FAD ratio was determined in cells [11,12,18] especially in brain tissues.

## 2. Experimental Methods

### 2.1. Materials

Alcohol dehydrogenase equine (lyophilized powder, recombinant, expressed in *E. coli*, Sigma Aldrich, Sofia, Bulgaria, lot 55689, CAS: 9031-72-5) was used in experiments. All samples were prepared in PBS buffer (pH = 7.2) fresh daily at 20 °C.

### 2.2. Composition Analysis

5 mg of lyophilized ADH were dissolved in 0.5 mL of sodium phosphate buffer containing 50 mM sodium phosphate, 150 mM NaCl, and 1 mM EDTA (pH = 8.0), prepurified by centrifugation at 16,000× *g* for 10 min and further loaded on size-exclusion column (GE HiLoad 16/600 Superdex 200 pg). The column was preliminary equilibrated with sodium phosphate buffer. The chromatographic purification was implemented on Akta Purifier 10 (GE Healthcare) with a flow rate of 0.6 mL/min. The prepurified ADH was loaded by using a 0.5 mL loop. The protein fractions passed through the column and then were analysed by a UV detector at 280 nm. The fractions of 2 mL each were collected and further analysed by SDS-PAGE using a standard Laemmli protocol with 10% acrylamide gel. The gel staining was implemented with Coomassie brilliant blue dye G250.

The selected bands on the gel were further analyzed by peptide mass fingerprinting mass spectrometry (MS) to identify the proteins presented in bands. For this purpose, the bands were cut from gel, destained twice with a mixture of acetonitrile and ammonium bicarbonate, and then dehydrated with pure acetonitrile. After that in-gel tripsinolysis was implemented for 3 h at 37 °C and the digestion was stopped by the addition of trifluoroacetic acid. The solutions made were used for preparing samples for MALDI mass spectrometry under standard protocol. The samples were analysed using a mass spectrometer (FT-ICR MS 9.4 Tesla, Varian). Data analysis was implemented using Omega software and Mascot server. Only proteins identified significantly (*p* < 0.05) were shown in the results presented in Section 3.2.

### 2.3. Fluorescence Spectrally-Resolved and Time-Resolved Measurements

The experimental setup used was similar to that described in detail in our previous publications [23,24,25,26]. Briefly, fluorescence was excited by two-photon absorption of a molecular sample at 720 nm and at 840 nm with linearly polarized light. A femtosecond oscillator (Mai Tai HP DS, Spectra Physics) with pulse duration of 100 fs and repetition rate of 80.4 MHz was used as an excitation source. The laser beam polarization degree was better than 0.995, the polarization direction was controlled by a half waveplate. The laser beam was expanded by a telescope to a diameter of 4 mm and then focused onto the center of a quartz cuvette containing the molecular sample. Average laser beam intensity on the cuvette was about 600 mW.

The fluorescence was collected in the direction perpendicular to the laser beam. In the spectrally-resolved experiments the fluorescence was detected at the magic angle condition to produce polarization-insensitive fluorescence. The fluorescence beam was spectrally selected by a monochromator (MDP-12, LOMO) and then recorded by a photomultiplier (H10682-01, Hamamatsu) operated in the photon counting mode. The photomultiplier output was analyzed by a TCSPC system (PicoHarp 300, PicoQuant) to produce fluorescence decay curves $I_{tot}\left(t\right)$ at several wavelengths in the spectral range of 390–560 nm with a step of 10 nm. Then mean fluorescence intensity $I_{tot}$ was calculated by integration of fluorescence decay signals as a function of fluorescence wavelength.

In the time-resolved experiments the polarization components of the fluorescence intensity parallel $I_{\|}$ and perpendicular $I_{⊥}$ to the excitation polarization plane were separated by a Glan prism and then simultaneously detected by two ultrafast avalanche photodetectors (SPAD, $PD-050-CTC, MPD) operated in a photon-counting mode. The fluorescence intensity was detected within the spectral bands of 426–446 nm or 460–480 nm selected by interference bandpass filters. The electric pulses from the photodetectors were analyzed by the TCSPC system and collected during 30 min with a time bin of 4 ps. The obtained signals were combined according to Equation (3) to produce polarization-insensitive fluorescence decay curves $I_{tot}\left(t\right)$.

### 2.4. Experimental Data Processing

The recorded orthogonal fluorescence polarization components $I_{\|}$(*t*) and $I_{⊥}$(*t*) can be presented by the expressions [27]:

(1) $$I_{\|}\left(t\right)=G\int_{0}^{t}\mathrm{IRF}\left(t^{\prime }\right)I_{l}\left(t-t^{\prime }\right)\left[1+2r_{l}\left(t-t^{\prime }\right)\right]dt^{\prime }$$

(2) $$I_{⊥}\left(t\right)=\int_{0}^{t}\mathrm{IRF}\left(t^{\prime }\right)I_{l}\left(t-t^{\prime }\right)\left[1-r_{l}\left(t-t^{\prime }\right)\right]dt^{\prime },$$

where $I_{l}\left(t-t^{\prime }\right)$ and $r_{l}\left(t-t^{\prime }\right)$ are isotropic and anisotropy intensity components, respectively, the subscript index *l* refers to the linear polarization of the excitation laser beam, IRF(*t*) is an instrumental response function, and *G* is a ratio of sensitivity of two fluorescence detection channels.

Polarization-insensitive fluorescence intensity $I_{tot}\left(t\right)$ was calculated from the experimentally determined polarization components $I_{\|}$(*t*) and $I_{⊥}$(*t*) in Equations (1) and (2) according to:

(3) $$I_{tot}\left(t\right)=\frac{\frac{1}{G}I_{\|}\left(t\right)+2I_{⊥}\left(t\right)}{3},$$

The function $I_{tot}\left(t\right)$ in Equation (3) is completely equivalent to the fluorescence intensity that can be recorded within magic-angle experimental geometry [2]. The function $I_{tot}\left(t\right)$ was approximated as a sum of exponentials [12,27,28,29]:

(4) $$I_{tot}\left(t\right)=I_{0}\sum_{i=1}^{n}a_{i}\;\exp\left(-\frac{t}{\tau_{i}}\right),$$

where $I_{0}$ is a time-independent initial fluorescence intensity, $\tau_{i}$ are decay times, and $a_{i}$ are corresponding weighting coefficients that are normalized to unity: $\sum_{i}a_{i}=1$.

The fitting procedure and estimation of errors were similar to that described in detail in our recent publications [27,30]. As in the conditions of our experiments the number of fluorescence photon counts was relatively small, the maximum likelihood method with the Poisson function $\chi^{2}$ [31,32] was used in the fit procedure. Minimization of the cost function was carried out by stochastic optimization algorithm using differential evolution implemented in *SciPy*. The IRF shape was determined experimentally by recording laser pulses at 436 and 470 nm scattered from a fiber sample. The effective FWHM of the IRF was determined to be about 120 ps at 436 nm and and 50 ps at 470 nm.

## 3. Results

### 3.1. Two-Photon Excited Fluorescence Spectrum of the Recombinant Equine ADH Reagent

**Figure 1:**
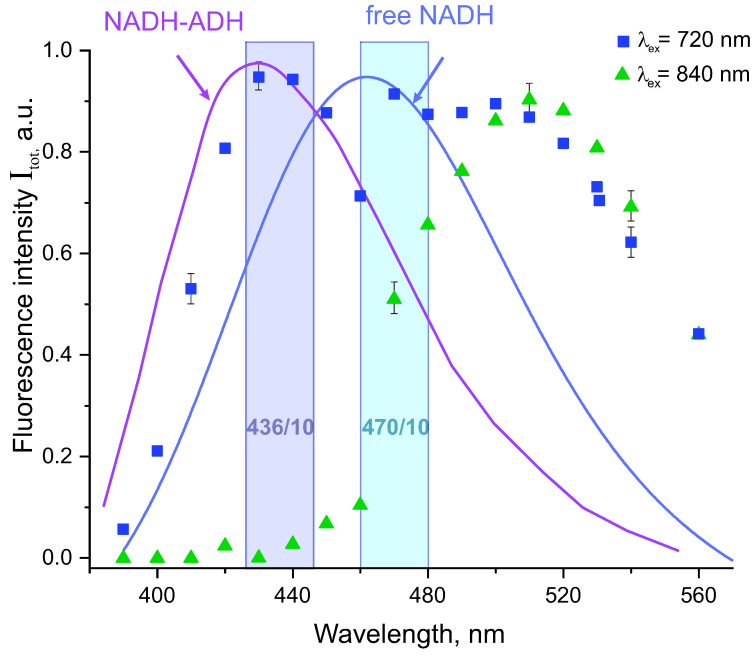
Fluorescence spectrum of the recombinant equine ADH reagent under two-photon excitation at 720 nm (blue squares) and 840 nm (green triangles). Blue curve is fluorescence spectrum of free NADH in buffer solution under two-photon excitation at 720 nm recorded in this paper. Violet curve is the fluorescence spectrum of NADH-ADH in buffer solution under one-photon excitation at 330 nm from Ref. [33]. Violet and blue rectangles represent the transmission bands of the bandpass filters 436/10 and 470/10, respectively that were used in time-resolved fluorescence experiments. The fluorescence intensities are normalized to unity.

Fluorescence spectra of the recombinant equine ADH reagent recorded under two-photon excitation at 720 nm and 840 nm are shown in Figure 1 with blue and green symbols, respectively. The experimental error bars in Figure 1 were determined using the procedure described in detail in our previous paper [27].

A wide fluorescence band with two maxima at 425 nm and 510 nm given with blue squares in Figure 1 was observed under excitation at 720 nm, while the fluorescence band observed under excitation at 840 nm and given with green triangles was noticeably narrower and had a single maximum at 515 nm. The fluorescence spectrum of free NADH in buffer solution under two-photon excitation at 720 nm determined in this paper is shown in Figure 1 with a blue line. The fluorescence spectrum of a purified ADH (from Boehringer Mannheim Co., Mannheim, Germany) in buffer solution under one-photon excitation at 330 nm reported almost thirty years ago by Gafni et al. [33,34] is also shown in Figure 1 with a violet line. One can see that the fluorescence maximum at 425 nm observed under excitation at 720 nm coincides perfectly with the maximum of NADH-ADH fluorescence spectrum reported by Gafni et al. [33,34]. Also, the fluorescence maximum observed under excitation at 840 nm roughly coincided with the long-wavelength maximum of the fluorescence band recorded under excitation at 720 nm. These data suggest that fluorescence maximum at 425 nm observed in our experiment under excitation at 720 nm related mostly to the fluorescence of NADH-ADH complex although free NADH existed in solution could also contribute to the recorded signal.

It should be pointed out that the red edge of the fluorescence signals in Figure 1 with maximum at 515 nm could not arise from NADH because two-photon excitation energy at 840 nm could not reach the absorption band neither in free, nor in bound forms of NADH [6,26,35]. Therefore, other chromophores that existed in the reagent were responsible for the fluorescence band with a maximum at 515 nm. This fact was not surprising as the commercial reagent of equine ADH (Sigma Aldrich) used in our experiments was an active recombinant enzyme expressed in *E. coli* and the purification procedure could not guarantee the absence of contaminating *E. coli* enzyme complexes other than ADH in the reagent [36]. Thereforte the analysis of the ADH reagent composition and characterisation of these fluorescent complexes were important this paper aims.

### 3.2. Composition Analysis of the Reagent of Recombinant Equine ADH through SDS-PAGE and FPLC

**Figure 2:**
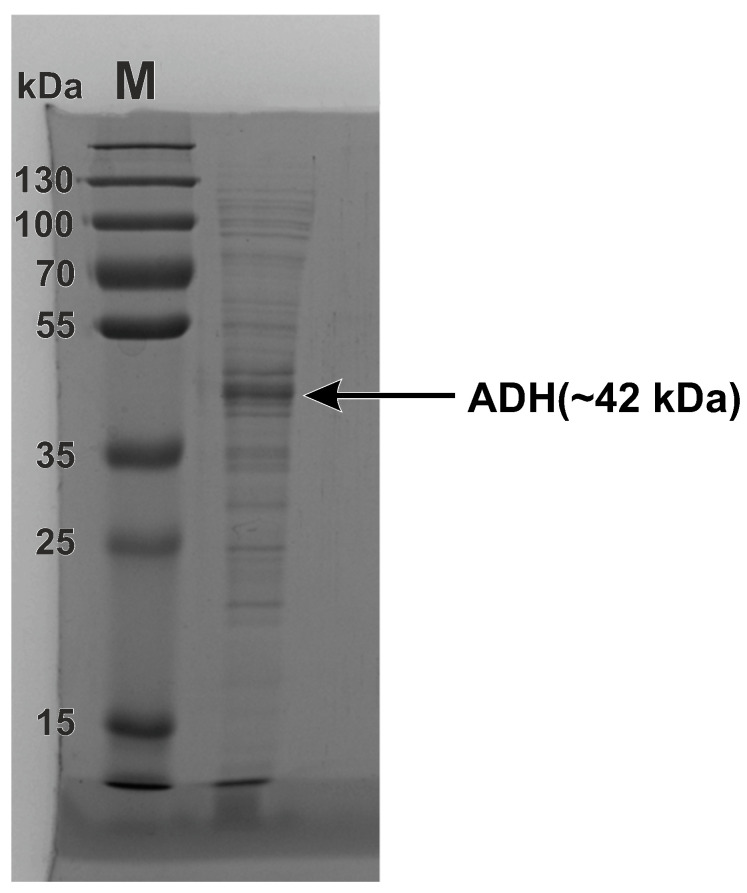
SDS-PAGE of the ADH reagent. The first lane is a molecular weight marker M.

The composition analysis of the reagent of recombinant equine ADH was performed through SDS-PAGE and FPLC. On the first step proteins in the reagent were separated under denaturing conditions using SDS-PAGE. As can be seen in Figure 2, in addition to the major band (42 kDa) related to the ADH monomer many additional bands of *E. coli* proteins were also recorded. Then, for characterization of the contaminant proteins the ADH preparation was subjected to FPLC.

**Figure 3:**
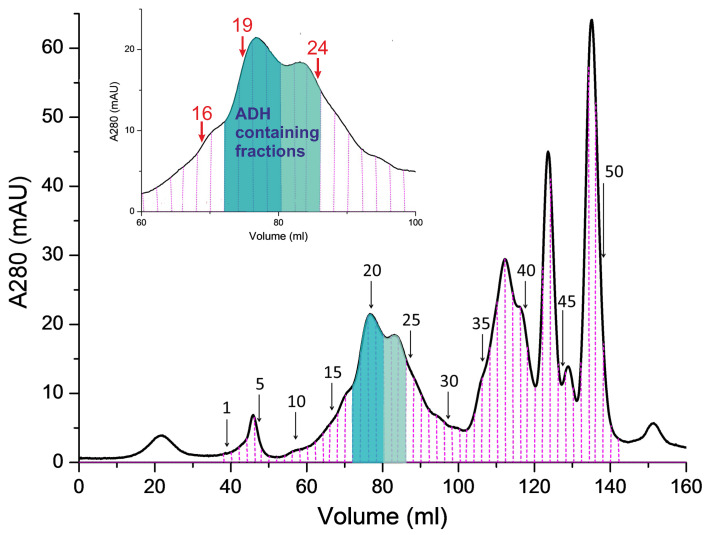
Chromatogram of the ADH reagent. The fractions with molecular weight close to ADH dimer are shown in the inset in an expanded scale. The fractions 18–21 that exhibited fluorescence under excitation at 720 nm and 840 nm are colored with dark green. The fractions 22–24 that exhibited fluorescence only under excitation at 720 nm, but not 840 nm are colored with light green.

The recorded chromatogram of the ADH reagent is shown in Figure 3 where vertical pink dash lines denote the boundaries of different protein fractions. Several intensive peaks shown in the chromatogram in Figure 3 indicate the existence of a large number of contaminating proteins in ADH reagent. Some of these fractions are labelled with black numbers with arrows. The fractions 15–30 referring to the molecular weights in the range of 70–100 kDa are expected to contain equine ADH dimers with molecular weight of 80 kDa. The fractions 35–40 referring to the molecular weights in the range of 35–50 kDa are expected to contain equine ADH monomers. The fractions 1–5 refer to the aggregates with molecular weight up to 150 kDa and the fractions 45–50 correspond to relatively small proteins.

Light emission properties of the fractions shown in Figure 3 were tested as follows. Each fraction was illuminated successively at 720 nm and 840 nm and the fluorescence intensity was recorded in the narrow spectral ranges selected by the dichroic bandpass filters 436/10 nm and 460/10 nm with transmission bands shown by violet and blue rectangles in Figure 1. It was found that only fractions 18–21 and 22–24 exhibited fluorescence under excitation moreover the recorded fluorescence intensity was much higher in the former than in the latter fraction group. In the following the group of the fractions 18–21 will be referred to as fraction A and the group of the fractions 22–24 will be referred to as fraction B. Integral fluorescence intensities of the fractions A and B are collected in Table 1.

As can be seen in Table 1 the fractions B exhibited fluorescence only under excitation at 720 nm while the fluorescence intensity depended slightly on the bandpass filter used. The fractions A exhibited fluorescence under excitation at either 720 or 840 nm while the fluorescence intensity under excitation at 840 nm was about five times larger with the filter 470/10 than with the filter 436/10. This finding is in qualitative agreement with the fluorescence spectrum shown in Figure 1.

### 3.3. Time-Resolved Fluorescence Analysis of the ADH-Containing Fractions

**Figure 4:**
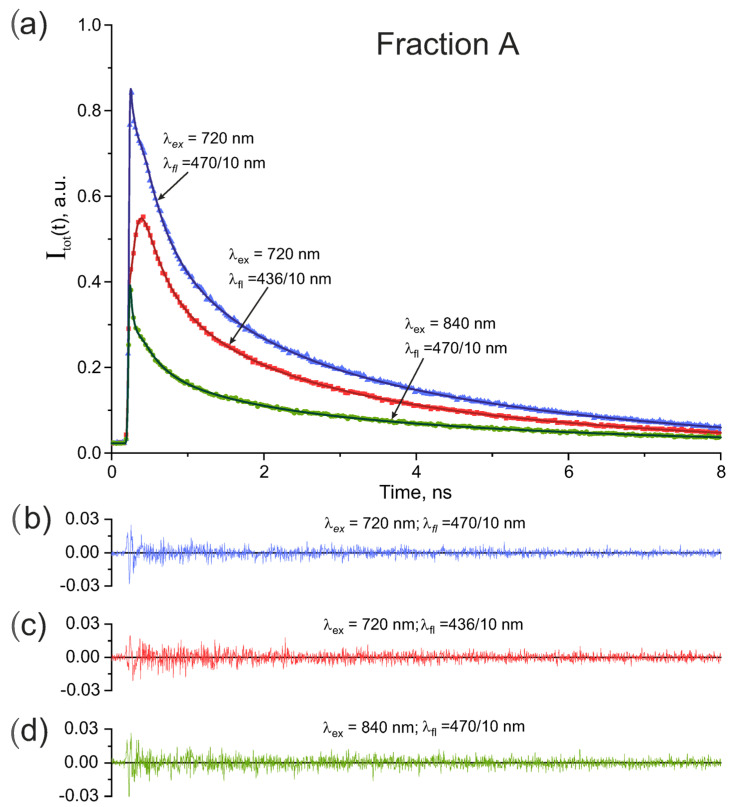
(**a**) Polarization-insensitive fluorescence decay curves in fraction A under two-photon excitation at 720 and 840 nm recorded through the fluorescence spectral windows of 470/10 nm and 436/10 nm. Experimental signals are given with blue, red, and green dots and solid lines are fits. (**b**–**d**) residuals used for control of the fit quality.

**Figure 5:**
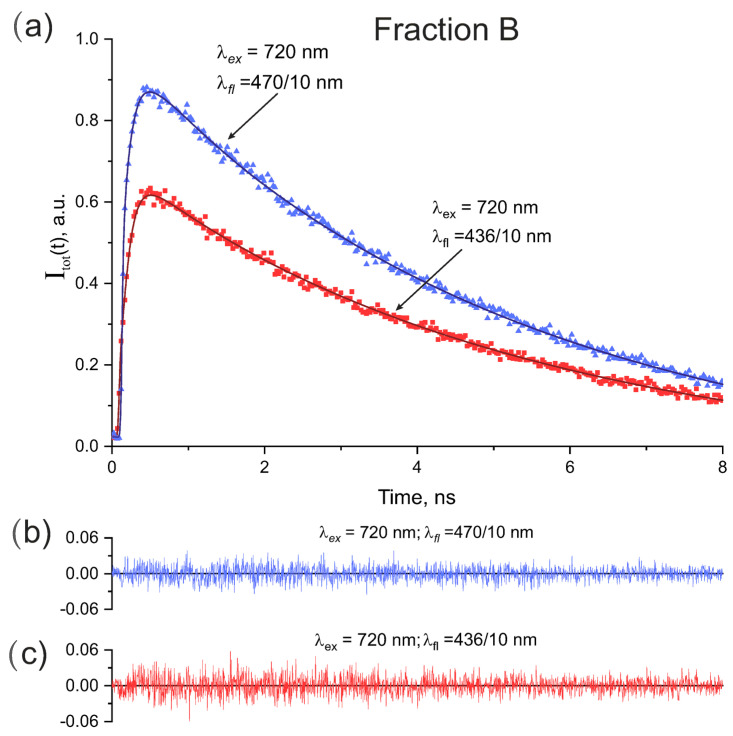
(**a**) Polarization-insensitive fluorescence decay curves in fraction B under two-photon excitation at 720 recorded through the fluorescence spectral windows of 470/10 nm and 436/10 nm. Experimental signals are given with blue and red dots and solid lines are fits. (**b**,**c**) residuals used for control of the fit quality.

Time-resolved fluorescence studies were carried out with the fractions A and B for deeper understanding of the fluorescence properties of the equine ADH reagent. As shown in Table 1 the fraction A could fluoresce under two-photon excitation at 720 nm and 840 nm, and the fraction B could fluoresce under two-photon excitation at 720 nm, but not under excitation at 840 nm. Fluorescence decay signals in the fractions A and B were detected in the spectral windows of 436/10 nm and 470/10 nm. Polarization-insensitive fluorescence decay curves $I_{tot}\left(t\right)$ obtained according to Equation (3) for the fractions A and B are presented in Figure 4 and Figure 5, respectively.

As can be seen in Figure 4 the intensities and shapes of the fluorescence signals in fraction A depended strongly on the excitation wavelengths and fluorescence spectral windows. At the same time the intensity of the fluorescence signals in fraction B in Figure 5 was sensitive to the fluorescence spectral windows however the signal shape remained practically the same.

The fluorescence decay signals in the Figure 4 and Figure 5 were approximated as a sum of exponentials in Equation (4) with one, two, three, or four terms and then decay times $\tau_{i}$ and weighting coefficients $a_{i}$ were determined from fit.

The results of approximation of the data in Figure 4 with two and four exponentials are presented in Table 2 and Table 3, respectively. It was found that for fraction A the four-exponential model in Table 3 gave the best fit quality for each excitation wavelengths and fluorescence spectral windows, while the double exponential model in Table 2 and three-exponential model (not shown) resulted in inappropriate $\chi^{2}$ values.

The results of approximation of the data in Figure 5 with one and two exponentials are presented in Table 4 and Table 5, respectively. As can be seen in Table 4 and Table 5 for fraction B the double-exponential model in Table 5 gave better fit quality at the fluorescence spectral window of 470/10 nm.

The best fit and residuals are given in Figure 4b–d and Figure 5b,c. The determined fluorescence decay times and corresponding weighting coefficients are collected in Table 3 and Table 5 for the fractions A and B, respectively.

As can be seen from Figure 4 and Figure 5 and Table 3 and Table 5 the time-resolved study of recorded fluorescence provided essential additional information on the fluorescing species in the equine ADH reagent as compared with fluorescence spectra in Figure 1. One can conclude that the fluorescence properties of the fractions A and B differed from each other dramatically and that the fraction A was found to be highly inhomogeneous containing not only ADH-bound NADH, but also other fluorescing compounds.

For more detailed characterisation of these compounds an additional composition analysis of several selected fractions shown in Figure 3 was performed by MS and found to be useful for understanding of the potential and limitations of optical methods as FLIM for separation of fluorescence signals from various natural fluorophores.

### 3.4. Composition Analysis of the ADH Fractions by MS

The detailed analysis of the selected fructions 16, 19, and 24 labelled in inset in Figure 3 was performed using SDS-PAGE and MS. As can be seen in Figure 3 the fractions 16 and 24 exhibited fluorescence and belonged to the fraction groups A and B, respectively, while the fraction 16 did not exhibit any fluorescence and was used for control.

**Figure 6:**
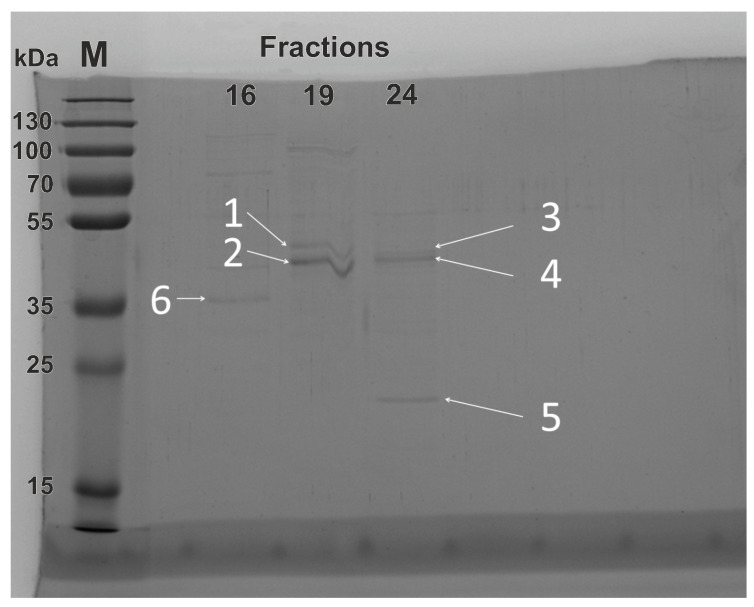
SDS-PAGE of fractions 16, 19, and 24. The first lane is the molecular weight marker M and white numbers 1–6 denote the bands that were analysed by MS.

The SDS-PAGE image of the fractions 16, 19, and 24 is presented in Figure 6. As can be seen in Figure 6 each of the 16, 19, and 24 fractions contained a number of various proteins. In particular in the first lane referred to fraction 16 the most intensive band at about 35 kDa is labelled as 6. The second lane referred to the fraction 19 contains two intensive bands at about 40–45 kDa that are labelled as 1 and 2 and several less intensive ones. The third lane referred to fraction 24 also contains several bands: two bands labelled as 3 and 4 that correspond to molecular weights of 40–42 kDa and a single band at 20 kDa labelled as 5. All proteins bands labelled by white numbers in Figure 6 were then analyzed by MS.

The results of MS analysis are shown in Table 6. As can be seen in Table 6 the fraction 19 contained the following mixture of enzymes: isocitrate dehydrogenase *E. coli* (ICDH, subunit molecular weight 46 kDa), phosphopyruvate hydratase *E. coli* (subunit molecular weight 46 kDa), serine hydroxymethyltransferase *E. coli* (SHMT, subunit molecular weight 46 kDa), and alcohol dehydrogenase equine (ADH, S subunit, subunit molecular weight 41 kDa). Three of these enzymes with the same subunit molecular weight of 46 kDa relate to band 1 in Figure 6 and the enzyme ADH with subunit molecular weight of 41 kDa relates to band 2. The fraction 24 contained a mixture of elongation factor Tu *E. coli* (subunit molecular weight 42 kDa, band 3 in Figure 6), alcohol dehydrogenase equine (ADH, S subunit, subunit molecular weight 41 kDa, band 4 in Figure 6), and superoxide dismutase [Mn] *E. coli* (subunit molecular weight 19.5 kDa, band 5 in Figure 6). The fraction 16 contained the enzyme Glyceraldehyde-3-phosphate dehydrogenase A (GAPDH, subunit molecular weight 37 kDa, band 6 in Figure 6). As can be seen in Table 6 the components corresponding to bands 4 and 5 were identified with relatively low significancy.

## 4. Discussion

### 4.1. Fluorescence Spectra Analysis

**Figure 7:**
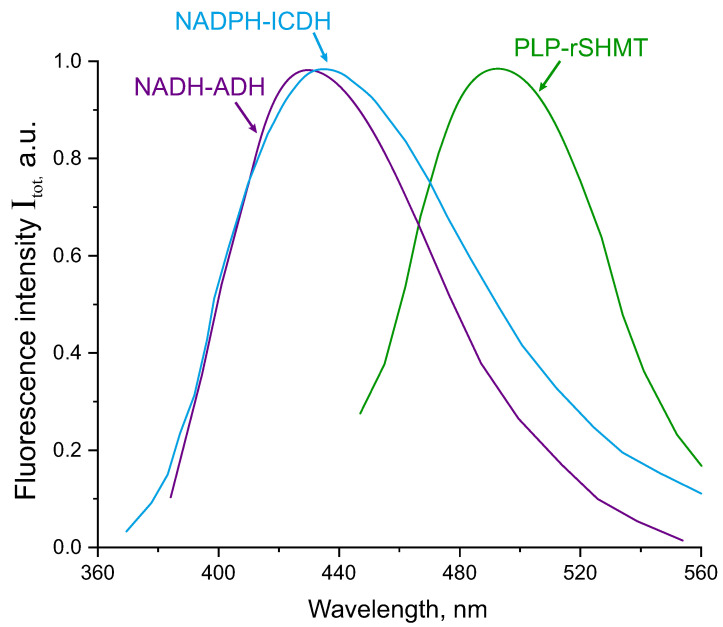
Fluorescence spectra of NADH-ADH [33] under excitation at 330 nm (violet curve), of PLP-rSHMT [39] (green curve), and of NADPH-ICDH (blue curve) [37].

As well known, absorption and emission spectra of NADPH bound to ICDH are almost identical with those of NADH bound to ADH [33,37]. Both complexes have absorption bands with a maximum at about 340 nm and could be two-photon excited at 720 nm in the conditions of our experiment, however they could not be excited at 840 nm. The fluorescence bands of NADH-ADH reported by Gafni et al. [33] and and NADPH-ICDH reported by Roman et al. [37] are shown in Figure 7. PLP-SHMT complex has two wide absorption peaks with maxima at 330 nm and 420 nm related to enolimine and ketoenamine tautomers of PLP, respectively [38,39,40,41] and therefore, it could be two-photon excited at either 720, or 840 nm in the conditions of our experiment. As known [39,40,41] the fluorescence properties of PLP are quite sensitive to microenvironment. When PLP is bound to bacterial SHMT it exhibits a fluorescence band with a maximum at about 505 nm [38,39,41] that is shown in Figure 7. Comparison between the data given in Figure 1 and Figure 7 manifests that the fluorescence band recorded in this paper under excitation at 840 nm and given in green triangles in Figure 1 agrees perfectly with that recorded in PLP-SHMT complex by Angelaccio et al. [39] and given with a green curve in Figure 7.

The existence of NADH-containing enzyme ADH, NADPH-containing enzyme ICDH, and PLP-containing enzyme SHMT in the ADH reagent under study was confirmed by direct composition analysis by SDS-PAGE and MS discussed in Section 3.4 and Section 4.3.

These data allow to characterise the shape of the experimental fluorescence spectra in equine ADH reagent shown in Figure 1. The fluorescence band with the maximum at 515 nm in Figure 1 observed under excitation at 720 nm and 840 nm arose most likely due to fluorescence of the PLP-SHMT complex. Also, as can be seen in Figure 7 the fluorescence bands of NADH-ADH and NADPH-ICDH almost fully overlap each other, therefore these complexes could not be separated spectroscopically and both could give contributions to the fluorescence band with the maximum at 425 nm in Figure 1. Therefore, under excitation at 720 nm both NADH-ADH and NADPH-ICDH complexes could contribute to the fluorescence band with the maximum at 425 nm and the PLP-SHMT complex contributed to the fluorescence band with the maximum at 515 nm. Also, under excitation at 840 nm the PLP-SHMT complex contributed to the fluorescence band with the maximum at 515 nm in Figure 1. These conclusions are supported by about five times difference between the fluorescence intensities recorded with the filters 470/10 and 436/10 under excitation at 840 nm and by comparable values of the fluorescence intensities recorded with each of the two filters under excitation at 720 nm in Table 1.

It is important that the contribution from the PLP-SHMT complex could be completely separated in the conditions of our experiment through two-photon excitation of the fluorescence at 840 nm and following fluorescence detection at about 515 nm because NADH-ADH and NADPH-ICDH complexes do not fluoresce under excitation at 840. Also, as shown in Figure 1 although two-photon excitation at 720 results in simultaneous excitation of the fluorescence of NADH-ADH, NADPH-ICDH, and PLP-SHMT complexes the contribution from the NADH-ADH and NADPH-ICDH complexes can be effectively separated using the 435/10 bandpass filter.

Moreover, it is important to note that absorption and fluorescence spectra of enzyme-bound PLP and FAD almost fully overlap [6,38]. Despite NADH concentration in cells is usually an order of magnitude higher than the concentration of other intrinsic fluorophores NADPH [42], FAD [3], and PLP [43] the latter can affect significantly on the observed fluorescence spectra. PLP is an active form of vitamin B6 that can be bound to many known cellular enzymes involved in the anabolic processes and neuronal metabolism [44]. As shown above PLP-SHMT complexes exist in detectable amount in the equine ADH reagent and can be effectively two-photon excited at wavelengths of 720 nm and 840 nm that are often used in autofluorescence studies of NADH and FAD in cells [4,45]. Therefore the contribution from bound PLP to fluorescence signal could mask the contribution from FAD that should be taken into consideration in FLIM studies where the NADH/FAD ratio was determined in cells [11,12,18] especially in brain tissues.

### 4.2. Analysis of Time-Resolved Fluorescence Intensity

As can be seen in Figure 4 and Figure 5 the shapes of the fluorescence decay curves depended dramatically on the fraction studied (A and B), on excitation wavelengths, and on bandpass filters used in the detection channel.

The fluorescence decay curves observed in fraction A are shown in Figure 4. Decay times and corresponding weighting coefficients calculated by fit of the experimental data in Figure 4 are collected in Table 3.

As shown in Section 4.3 and Section 4.1 the NADPH-ICDH and PLP-SHMT fluorescing complexes could exist in fraction A along with the NADH-ADH complex. All these complexes could absorb at excitation wavelengths of 720 and 840 nm used in our experiments and fluoresce within the spectral bands of 436/10 nm and 470/10 nm.

According to the discussion given in Section 4.3 the fluorescence decay times of the fraction A determined under excitation at 840 nm and presented in the first row in Table 3 were associated with PLP-SHMT complex. As known enzyme-bound PLP tautomers enolimine and ketoenamine absorb in a wide spectral range with maxima at 330 nm and 420 nm, respectively [38,40,43,46]. According to the report of Ahmed et al. [38] the reactions of PLP bound with β-subunit of tryptophan synthase with substrates and inhibitors often lead to alterations in recorded fluorescence spectrum and in fluorescence decay times. Similar alterations were observed under the change of solution pH. The fluorescence decay of PLP bound via a Schiff base in the β-subunit of the tryptophan synthase $\alpha_{2}\beta_{2}$ complex in the nanosecond time domain under excitation at 410 nm was studied by Vaccari et al. [47] who reported the fluorescence decay times of 2.9 ns and 0.9 ns of almost equal fractional intensity that were slightly affected by pH. More recently, Hill et al. [44] studied rapid fluorescence dynamic of PLP-valine complex in solution using the transient absorption spectroscopy and determined the decay times of about 1.16 ps, 63.8 ps, and 1.03 ns.

The fluorescence decay time of $\tau_{3}^{A}$ = 1.11 ns in the first row in Table 3 agrees perfectly with the times of 0.9 ns and 1.03 ns reported by Vaccari et al. [47] and by Hill et al. [44], respectively. However, the longest decay time of $\tau_{4}^{A}$ = 4.84 ns in the first row in Table 3 is about twice longer than the time of 2.9 ns reported by Vaccari et al. [47] for PLT-$\alpha_{2}\beta_{2}$ complex. This difference can likely be due to different proteins studied by Vaccari et al. [47] and in this paper.

Hill et al. [44] studied the ultrafast dynamics of PLP-valine complex by femtosecond transient absorption spectroscopy and reported the decay times of 0.001 ns and 0.06 ns. These data cannot be directly compared with short decay times of $\tau_{1}^{A}$ = 0.02 ns and $\tau_{2}^{A}$ = 0.20 ns given in the first row in Table 3 because the proteins bound with PLP were not the same in our experiments and in ref. [44] and because different experimental methods were utilized in this paper fluorescence experiments and by Hill et al. [44]. Temporal resolution of this paper experiments was affected by IRF of about 0.05 ns that decreased the accuracy of measurements of short decay times, although the IRF shape was carefully measured before experiments and then used in the global fit procedure as described in Section 2.4. The fluorescence decay times of $\tau_{1}^{A}$ = 0.02 ns and $\tau_{2}^{A}$ = 0.20 ns in the first row in Table 3 likely reflected the decay times 0.001 ns and 0.06 ns reported by Hill et al. [44]. The decay times given in the first row in Table 3 can be used for identification of the PLP-SHMT complex in FLIM experiments.

The second row in Table 3 shows the fluorescence decay times and weighting coefficients determined under two-photon excitation at 720 nm with the 436/10 nm bandpass filter inserted in the detection channel. In this case the PLP-SHMT fluorescence was excited, but was almost completely cut off by the 436/10 nm bandpass filter as shown in Figure 1 and Figure 7. According to the discussion in Section 4.3 and the data in Figure 1 and Figure 7 the fluorescence red decay curve $\lambda_{ex}=720$ nm, $\lambda_{fl}=436/10$ nm in Figure 4 was associated with NADH-ADH and NADPH-ICDH complexes, and also with free NADH and NADPH that could exist in solution because of the non-covalent bonding of the coenzymes with corresponding enzymes. The decay times and weighting coefficients shown in the second row in Table 3 are in the perfect agreement with the results reported in our recent publication [30].

The fluorescence decay times of $\tau_{2}^{A}$ = 0.33 ns and $\tau_{3}^{A}$ = 1.19 ns in the second row in Table 3 can be associated with free NAD(P)H. Fluorescence decay in free NADH and NADPH in solutions was studied by several groups [21,26,27,28,29,48,49], who reported biexponential decay with decay times of about 0.25 ns and 0.70 ns sensitive to microenvironmental conditions. We have recently suggested that the heterogeneity in the measured decay times in NADH is due to different charge distributions in *cis* and *trans* configurations of the nicotinamide ring resulting in different electrostatic field distributions and different non-radiative decay rates [27]. Note that the decay times $\tau_{2}^{A}$ and $\tau_{3}^{A}$ in the second row in Table 3 are somewhat longer than those determined in water and in organic solutions [21,26,27,28,29]. This could be due to lowering of solution polarity in close proximity of enzymes in the conditions of our experiments.

The fluorescence decay times $\tau_{1}^{A}$ = 0.08 ns and $\tau_{4}^{A}$ = 4.26 ns in the second row in Table 3 can be associated with fluorescence from NADH-ADH and NADPH-ICDH complexes. The decay time of $\tau_{4}^{A}$ = 4.26 ns is in a perfect agreement with the decay times of 4.2 ns reported for NADH-ADH complex by Gafni et al. [34], 4.8 ns reported by Ladokhin and Brand [35], and 4.85 nm reported by Gorbunova et al. [30] however it is somewhat longer than the decay times of 2.9 ns and of 3.16 ns reported by König et. al. [50] and by Piersma et al. [51]. Leben et al. [20] have recently reported the fluorescence decay times of 2.6 ns for NADH-ADH complex and 2.17 ns for NADPH-ICDH complex in buffer solution.

A significant dispersion of the experimental values of the decay time related to the time $\tau_{4}^{A}$ in Table 3 in NADH-ADH and NADPH-ICDH complexes reported elsewhere can be associated with different microenvironmental conditions: refractive index, solvent polarity, pH, ion concentration, etc. [20] in different experiments, and also with contributions of possible unaccounted fluorophores with overlapping absorption and fluorescence spectra that could exist in enzymatic solutions. We believe that contributions from NADH-ADH and NADPH-ICDH complexes could not be separated in the conditions of our experiments, although the results of mass spectrometry analysis given in Section 3.2 undoubtedly indicate the presence of both NADH-containing and NADPH-containing enzymes in the molecular sample used.

Yaseen et al. [14] used four exponential function for analysis of FLIM data obtained from cortical surface in vivo under two-photon excitation at 740 nm and determined fluorescence decay times of 0.406 ns and 1.102 ns which were attributed to free NADH, and two nanosecond decay time of 2.28 ns and 4.487 ns which were attributed to bound NADH species. Interestingly, that the fluorescence decay times presented in the second row of Table 3 are similar to those determined in cells by Yaseen at al [14].

The decay time of $\tau_{1}^{A}$ = 0.08 ns in the second row in Table 3 is in a good agreement with that reported earlier by Ladokhin and Brand [35] in NADH-containing ADH, by Piersma et al. [51] in nicotinoprotein alcohol dehydrogenase (np-ADH), and by Vishwasrao et al. [12] in cells and was associated with the electron transfer in the NADH excited state occurred due to interaction with ADH binding site. Very similar decay time value was recently reported by Gorbunova et al. [30] who also developed a theoretical model (see also a quantum mechanical approach in Ref. [52]) suggesting that the decay time $\tau_{1}^{A}$ reflects reversible interactions either within ADH binding sites, or between two NADH molecules bound with an ADH dimer. As shown below the decay time $\tau_{1}^{A}$ that was usually not taken into account in FLIM experiments [11,20] is an important characteristic of NADH fluorescence allowing for separation NADH from other enzyme-bound fluorophores.

The third row in Table 3 shows the fluorescence decay times and weighting coefficients determined under two-photon excitation at 720 nm with the 470/10 nm bandpass filter inserted in the detection channel. In this case all fluorescence compounds mentioned above: PLP-SHMT, NADH-ADH, NADPH-ICDH, free NADH and NADPH could be excited to give contributions to the observed fluorescence signal shown in Figure 4 with a green curve. As can be seen in Figure 3 the blue and green fluorescence decay curves contained much sharper peaks at short decay times then the red decay curve. This drastic difference can also be seen in Table 3 where the decay times and corresponding weighting coefficients in the first and third lines describing the decay of PLP-containing enzyme mixtures show significant contributions from the shortest decay times of 0.02 and 0.03 ns in comparison with PLP-lacking enzyme mixtures in the second row where the shortest decay time of 0.08 ns is several times longer. These results suggest that the fluorescence decay time $\tau_{1}^{A}\leq$ 0.02 ns is an important feature of PLP-SHMT complex that allows to separate them from enzyme-bound NAD(P)H without spectral resolution of fluorescence.

This conclusion is likely also true for separation of PLP-SHMT from FAD-enzyme complex. As reported by Chorvat and Chorvatova [45] the fluorescence dynamics of FAD complexes with enzymes in various cell examined by TCSPC technique does not contain as short fluorescence decay times as $\tau_{1}^{A}$ = 0.02 ns shown in the first row in Table 3. Therefore, although the fluorescence spectra of PLP-SHMT complex and FAD-enzyme complexes practically overlap [6,38], these complexes still can be distinguished from each other by comparing the shortest decay times of tens of picoseconds that can still be determined with TCSPC method. Therefore, the data given in Figure 4 and in Table 3 suggests that time-resolved fluorescence measurements allow to separate PLP-SHMT and FAD complexes with enzymes.

The fluorescence decay curves observed in the fraction B are shown in Figure 5. As can be seen in Table 5, in this case one- or two-exponential models were used to fit the fluorescence decay signals with sufficient accuracy. As shown in Table 6 the results of mass spectrometry analysis indicated that fraction B contained only NADH-ADH fluorescing complex. An apparent contradiction between the fluorescence parameters given in Table 5 and those in the second row in Table 3 was due to fairly small ADH concentration in the fraction B (score of 47 with critical score of 70 in Table 6) that led to relatively small number of peak photon counts in recorded signals and decreased signal-to-noise ratio. Moreover, as can be seen in Figure 3 the ADH enzymes in the fraction B had somewhat smaller masses than those in the fraction A and therefore the fluorescence properties of these two groups could be different. As can be seen in Table 5, the fluorescence signals recorded with 470/10 nm and 436/10 nm bandpass filters both contained the long decay time of about 5.2 ns that could be associated with the NADH-ADH bound form. The fluorescence signal recorded with 470/10 nm bandpass filter contained also the second decay time of 0.43 ns that could be associated with a mean value of the decay times of free NADH forms. At the same time, the short decay time of 0.08 ns in the second row in Table 3 does not exist in Table 5 as it could not be resolved in the conditions of relatively small number of peak photon counts in experiment with the fraction B.

Therefore, the separation of PLP-SHMT complex from NADH- and FAD-enzyme complexes mentioned above is likely possible only at relatively high number of peak photon counts in recorded fluorescence signals.

### 4.3. Composition Analysis

As was shown in Section 3.2 the recombinant equine ADH reagent under study contained a number of fluorescing compounds in fractions A and B.

According to the results of the mass-spectrometry analysis shown in Table 6 the fraction A contained four enzymes: ICDH, ENO1, SHMT, and ADH three of those can form complexes with fluorescing coenzymes. These are noncovalently bound complexes NADH-ADH and NADPH-ICDH [33,37] and the complex PLP-SHMT, where PLP is a covalently bound pyridoxal 5-phosphate coenzyme [38,39,40,41]. The reagent ENO1 does not form a complex with a fluorescent coenzyme [53] and does not seem to fluoresce at the excitation wavelengths used in these experiments. As can be seen in Table 6 the fraction B contained three enzymes: EF-Tu, SOD, and ADH. The first two of them likely do not fluoresce at the excitation wavelengths used in these experiment [54,55] and the third one, ADH could contain the coenzyme NADH that fluoresces under two-photon excitation at 720 nm but could not fluoresce under excitation at 840 nm.

## 5. Conclusions

The composition analysis of a commercial reagent of equine NADH-ADH expressed and purified from *E. coli* by means of gel electrophoresis, chromatography, and mass spectrometry indicated contributions also from NADPH-ICDH and PLP-SHMT fluorophores. Spectral- and time-resolved studies of the reagent fluorescence under two-photon excitation at 720 nm and 840 nm demonstrated the potential and limitations of FLIM for separation of fluorescence signals from coenzymes NADH, NADPH, and FAD in presence of other fluorophores. As shown, contribution of PLP-SHMT complex could be separated from that of NADH-ADH and NADPH-ICDH complexes through two-photon excitation of fluorescence at 840 nm. Also, the contribution from PLP-SHMT complex under two-photon excitation at 720 nm could be almost completely damped by using a 436/10 bandpass filter in the fluorescence channel. Analysis of time-resolved fluorescence signals demonstrated the existence of a relatively short fluorescence decay time of about 0.02 ns in PLP-containing enzyme mixtures and several times longer decay time of about 0.08 ns in PLP-lacking enzyme mixtures. This finding suggests that the fluorescence decay time 0.02 ns is an important feature of PLP-SHMT complex that allows to separate them from enxyme-bound NAD(P)H without spectral resolution of fluorescence. It is even more important that determination and analysis of short fluorescence decay times of several tens of picoseconds can allow to separate PLP-containing enzymes from enzyme-bound FAD although their fluorescence spectra practically overlap each other. At the same time NADH-ADH complex could not be optically separated from NADPH-ICDH complex in our experimental conditions although the FPLC and mass spectrometry analysis undoubtedly indicated the presence of both enzymes in the molecular sample used. The results obtained can be used for interpretation of fluorescence signals from a mixture of enzyme-bound fluorophores and should be taken into consideration in determination of the NADH/FAD ratio with FLIM in cells.

## Figures and Tables

**Table 1:** Fluorescence intensities in the fractions A and B under excitation at 720 nm and 840 nm recorded within the spectral ranges of 436±10 nm and 470±10 nm.

Excitation	Fluorescence Filter	$I_{\mathrm{tot}}$, a.u.	$I_{\mathrm{tot}}$, a.u.
nm	nm	Fraction A	Fraction B
720	436/10	0.71 ± 0.03	0.31 ± 0.06
720	470/10	1.00 ± 0.03	0.43 ± 0.07
840	436/10	0.08 ± 0.09	–
840	470/10	0.39 ± 0.05	–

**Table 2:** Fluorescence decay times $\tau_{i}^{A}$ and weighting coefficients $a_{i}$ determined in the fraction A using two-exponential model.

Exc, nm	Fluo, nm	$\tau_{1}^{A}$, ns ($a_{1}$)	$\tau_{2}^{A}$, ns ($a_{2}$)	$\chi^{2}$
840 nm	470/10	0.14 (0.73)	3.24 (0.27)	5.41
720 nm	436/10	0.23 (0.65)	3.00 (0.35)	5.00
720 nm	470/10	0.19 (0.69)	3.18 (0.31)	8.87

**Table 3:** Fluorescence decay times $\tau_{i}^{A}$ and weighting coefficients $a_{i}$ determined in the fraction A using four-exponential model.

Exc, nm	Fluo, nm	$\tau_{1}^{A}$, ns ($a_{1}$)	$\tau_{2}^{A}$, ns ($a_{2}$)	$\tau_{3}^{A}$, ns ($a_{3}$)	$\tau_{4}^{A}$, ns ($a_{4}$)	$\chi^{2}$
840 nm	470/10	0.02 (0.62)	0.20 (0.18)	1.11 (0.09)	4.84 (0.09)	1.18
720 nm	436/10	0.08 (0.43)	0.33 (0.19)	1.19 (0.17)	4.26 (0.21)	1.15
720 nm	470/10	0.03 (0.44)	0.22 (0.27)	1.14 (0.13)	4.68 (0.15)	1.19

**Table 4:** Fluorescence decay times $\tau_{i}^{B}$ and weighting coefficients $a_{i}$ determined in the fraction B using one-exponential model.

Exc, nm	Fluo, nm	$\tau_{1}^{B}$, ns ($a_{1}$)	$\chi^{2}$
720 nm	436/10	5.14 (1.00)	1.16
720 nm	470/10	4.93 (1.00)	2.08

**Table 5:** Fluorescence decay times $\tau_{i}^{B}$ and weighting coefficients $a_{i}$ determined in the fraction B using double-exponential model.

Exc, nm	Fluo, nm	$\tau_{1}^{B}$, ns ($a_{1}$)	$\tau_{2}^{B}$, ns ($a_{2}$)	$\chi^{2}$
720 nm	436/10	-	5.14 (1.00)	1.16
720 nm	470/10	0.43 (0.09)	5.20 (0.91)	1.08

**Table 6:** Results of mass spectrometry analysis of the bands 1–6.

Fraction (Band)	Identifier	Protein, Weight	Organism	Score	Critical Score
19 (1)	AAN06339.1	ICDH, 92 kDa	*E. coli*	137	98
	(NCBIprot)				
19 (1)	WP_053270889.1	ENO1, 92 kDa	*E. coli*	149	98
	(NCBIprot)				
19 (1)	GLYA_ECO24	SHMT, 92 kDa	*E. coli*	71	70
	(SwissProt)				
19 (2)	ADH1S_HORSE	ADH, 82 kDa	Eq. caballus	82	70
	(SwissProt)				
24 (3)	QKN32973.1	EF-Tu, 84 kDa	*E. coli*	194	98
	(NCBIprot)				
24 (4)	ADH1S_HORSE	ADH, 82 kDa	Eq. caballus	47	70
	(SwissProt)				
24 (5)	WP_097500072.1	SOD, 79 kDa	*E. coli*	57	98
	(NCBIprot)				
16 (6)	G3P1_ECO57	GAPDH, 142 kDa	*E. coli*	108	70
	(SwissProt)				

Abbreviations in column 3: ICDH: Isocitrate dehydrogenase; ENO1:Phosphopyruvate hydratase; SHMT: Serine hydroxymethyltransferase; ADH: Alcohol dehydrogenase S chain; EF-Tu: Elongation factor Tu; SOD: Superoxide dismutase; GAPDH: Glyceraldehyde-3-phosphate dehydrogenase A.

## Data Availability

Not applicable.

## Abbreviations

The following abbreviations are used in this manuscript:

NAD(P)H	Nicotinamide adenine dinucleotide (phosphate)
FAD	Oxidized flavine-adenine dinucleotide
FMN	Flavin mononucleotide
FLIM	Fluorescence-lifetime imaging
TCSPC	Time-correlated single photon counting
ADH	Alcohol dehydrogenase
ICDH	Isocitrate dehydrogenase
PLP	pyridoxal 5-phosphate
SHMT	Serine hydroxymethyltransferase
SDS-PAGE	Sodium dodecyl sulfate–polyacrylamide gel electrophoresis
MALDI	Matrix-assisted laser desorption/ionization
ENO1	Phosphopyruvate hydratase
EF-Tu	Elongation factor Tu
GAPDH	Glyceraldehyde-3-phosphate dehydrogenase A
SOD	Superoxide dismutase
FPLC	Fast protein liquid chromatography
